# MitraClip for the treatment of heart failure with mitral regurgitation: A cost-effectiveness analysis in a Chinese setting

**DOI:** 10.3389/fcvm.2022.970118

**Published:** 2022-11-11

**Authors:** Wengang Xia, Kangning Han, Yake Lou

**Affiliations:** ^1^Department of Cardiology, Zigong Third People's Hospital, Zigong, China; ^2^Department of Cardiology, Beijing Anzhen Hospital, Capital Medical University, Beijing, China; ^3^Department of Cardiology, The Second Affiliated Hospital of Chongqing Medical University, Chongqing, China

**Keywords:** MitraClip, heart failure, mitral regurgitation, transcatheter mitral valve repair, cost-effectiveness analysis

## Abstract

**Background:**

Heart failure (HF) with mitral regurgitation is associated with decreased survival. Guideline-directed medical therapy and transcatheter edge-to-edge repair (TEER) are the main options for HF patients with severe mitral regurgitation who are considered high-risk or prohibitive. To date, there have been no studies investigating the cost-effectiveness of MitraClip vs. optimal medical therapy (OMT) in a Chinese setting.

**Methods:**

A combined decision tree and Markov model were developed to compare the cost-effectiveness MitraClip vs. OMT with a lifetime simulation. The primary outcome was the incremental cost-effectiveness ratio (ICER), which represented incremental costs per quality-adjusted life-year (QALY). The willingness-to-pay (WTP) threshold was set three times of per capita gross domestic product (GDP) in China in 2021, which was 242,928 CNY. MitraClip would be considered cost-effective if the ICER obtained was lower than the WTP threshold. Otherwise, it would be not considered cost-effective. One-way sensitivity and probabilistic sensitivity analyses were performed to validate the robustness of the results.

**Results:**

After a simulation of the lifetime, the overall cost for a patient in the MitraClip cohort was 423,817 CNY, and the lifetime cost in the OMT was 28,369 CNY. The corresponding effectiveness in both cohorts was 2.32 QALY and 1.80 QALY per person, respectively. The incremental cost and increment effectiveness were 395,448 CNY and 0.52 QALY, respectively, and the ICER was 754,410 CNY/QALY. The ICER obtained was higher than the WTP threshold. Sensitivity analysis validated our finding.

**Conclusion:**

MitraClip provided effectiveness but with more costs compared with OMT, and the incremental cost-effectiveness ratio obtained was higher than the WTP threshold. MitraClip was considered not cost-effective in Chinese HF patients with secondary mitral regurgitation.

## Introduction

Heart failure (HF), a clinical consequence arising from various causes, accounts for at least 20% of hospital admissions among patients older than 65 years (1). Uncorrected valvular diseases, such as mitral regurgitation (MR), often cause diastolic HF. The remodeling of the left ventricle (LV) caused by ischemic or dilated cardiomyopathy leads to displacements of papillary muscles and tethering of leaflets, contributing to secondary MR (2).

Studies have suggested that there is an association between MR and decreased survival in HF patients (3). MR could deteriorate LV function, resulting in adverse clinical outcomes due to a progression of LV remodeling (2). The coexistence of MR and HF significantly worsens the prognosis, and MR is an important therapeutic target for those patients (4). However, surgery is not recommended in patients with severe MR who are considered at high risk or prohibitive. For those patients, guideline-directed medical therapy (MT) and transcatheter edge-to-edge repair (TEER) are the main options (5). MitraClip, the most commonly used device of TEER, is significantly safer than surgery and improves the New York Heart Association functional class and overall survival rates (6, 7).

Since the global problem of HF is growing, the economic burden needs to be addressed. China has recently experienced an increase in HF prevalence of about 2% in recent years, with an estimated 8–10 million patients (8). In 2012, the medical security system of China faced a cost of approximately $5.4 billion related to HF (9). Although TEER is more effective than MT, the relatively high cost has hampered its widespread clinical use in China. Even in developed countries, MitraClip is highly expensive among cardiac therapies. Therefore, evaluating the cost-effectiveness of MitraClip is important for the healthcare system in China.

## Materials and methods

### Aims and population

This study aimed to compare the cost-effectiveness of MitraClip plus optimal medical therapy (OMT) with OMT alone in Chinese HF patients with secondary MR from the perspective of a healthcare payer. The study was based on a Chinese setting, but the population was a hypothetical cohort with similar baseline characteristics to the patients in the COAPT trial (Cardiovascular Outcomes Assessment of the MitraClip Percutaneous Therapy for Heart Failure Patients With Functional Mitral Regurgitation) (7). In the cohort, the mean age was 72 years, 0.2% of patients had an NYHA classification of NYHA I, 39.0% of patients had an NYHA classification of NYHA II, 52.5% of patients had an NYHA classification of NYHA III, and 8.3% of patients had an NYHA classification of NYHA IV. The patients had a moderate-to-severe or severe secondary MR before enrollment and were randomized to receive MitraClip plus OMT or OMT alone. The inclusion and exclusion criteria of the study were similar to those in the COAPT trial and shown in the Supplementary material.

### Model overview

**Figure 1:**
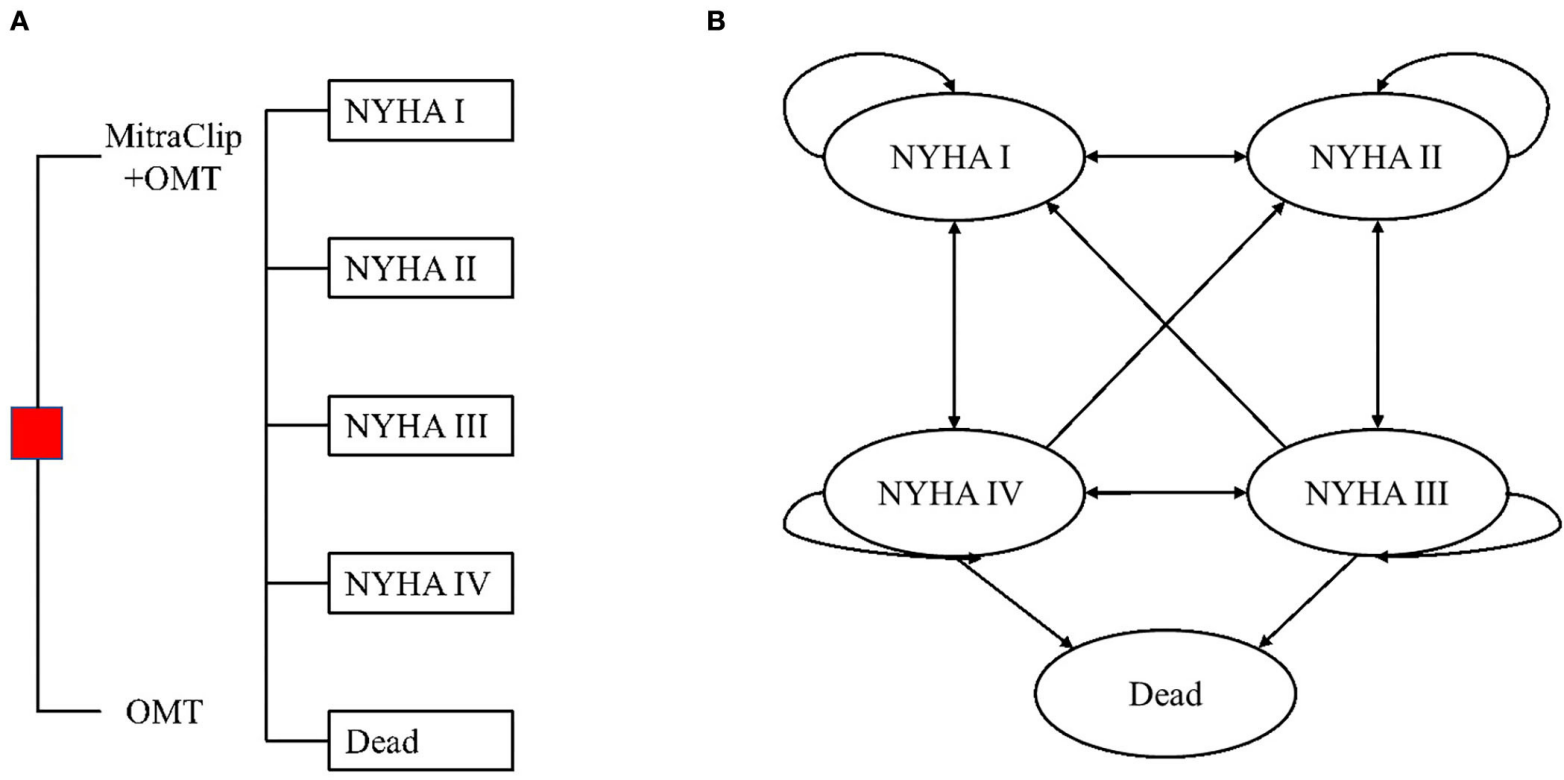
**(A)** Decision tree and **(B)** state transition diagram of the Markov model. OMT, optimal medical therapy. HF patients with moderate-to-severe or severe secondary MR were randomly allocated to receive MitraClip plus OMT or OMT alone. One month after the MitraClip procedure/OMT, patients would enter the Markov model and transition among these four NYHA classifications until they entered the terminal node of the dead.

The basic structure of the model consisted of two parts: one was a 30-day decision tree model, and another was a lifetime Markov model. In the 30-day decision tree model, Chinese HF patients with secondary MR were randomly allocated to receive the MitraClip procedure or OMT and would enter different NYHA classifications at the end of this stage. After this stage, the patients included would enter the Markov model with a cycle length of 1 month and a time horizon of a lifetime. In this model, patients would transition among four transition states, including NYHA I, NYHA II, NYHA III, and NYHA IV. If patients died during the cycle, they would enter the absorbed state of “dead,” meaning their cycle was finished. During the cycle, all the patients received OMT, and they also might have experienced HF hospitalization or no event. As the mean age in the study was 72 years and the time horizon was a lifetime, there would be 336 cycles, equal to 28 years, until the life of 100 years, which was far higher than the life expectancy in China. A half-cycle correction was employed in the Markov model to prevent the overestimation of effectiveness and cost. The details of the model are illustrated in Figure 1, which has been validated by another study (10).

### Input parameters

#### Transition probability

The transition probability in our model was mainly derived from the COAPT trial (7, 11). The 30-day outcome was directly extracted from the COAPT trial, and the transition probability in the Markov model was transformed from the COAPT trial to better represent the real efficacy of MitraClip *vs*. OMT. The transition probability in the COAPT trial was not reported in the published paper, but it was calculated by Estler et al. (10). The transition probability between NYHA classifications is presented in Table 1.

**Table 1:** Transition between NYHA classifications in MitraClip and OMT cohort.

** 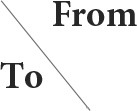 **	**NYHA I**	**NYHA II**	**NYHA III**	**NYHA IV**	**HF death**	**References**
**MitraClip/OMT**						
NYHA I	0.960/0.950	0.040/0.050	0/0	0/0	0/0	(7, 10)
NYHA II	0.005/0.010	0.945/0.940	0.050/0.040	0/0.010	0/0	(7, 10)
NYHA III	0/0	0.025/0.020	0.895/0.920	0.070/0.050	0.010/0.010	(7, 10)
NYHA IV	0/0	0/0	0/0	0.800/0.750	0.200/0.250	(7, 10)

NYHA, New York Heart Association; OMT, optimal medical therapy; HF, heart failure.

#### Costs

The cost of the MitraClip device and other MitraClip-related costs were accessed from a Chinese hospital (12), as there was no study on the cost of MitraClip in China. The cost of the MitraClip device was 322,000 Chinese Yuan (CNY) (equal to 49,922 USD, according to the average ratio of 6.45 in 2021), accounting for over 80% of the overall cost. Other MitraClip-related costs included procedure costs, nursing costs, ward costs, diagnosis costs, medicine costs, complication costs, etc. The cost of the MitraClip device and other MitraClip-related costs were only calculated in the MitraClip cohort, but the cost of OMT and HF hospitalization was calculated in both cohorts. The cost of OMT was derived from a study investigating the burden of HF in China, and the annual cost of OMT and cost of HF hospitalization were 5,138 CNY and 10,926 CNY, respectively (13). Regarding the cost of the MitraClip device and other MitraClip-related and HF hospitalization costs, a one-time cost was employed. However, for the cost of OMT, the annual cost was converted into the monthly cost and input into the model. All the costs were converted to the corresponding costs in China in 2021 using the Consumer Price Index (CPI) in China in the past few years (Table 2). The healthcare CPI in China from 2015 to 2021 were 1.027, 1.038, 1.06, 1.043, 1.024, 1.018, and 1.004, separately (23).

**Table 2:** Input parameters of decision tree and Markov model.

**Parameters**	**Base**	**Range**	**Distribution**	**References**
**Cost of MitraClip related (CNY)**				
Device	322,000	161,000–386,400	γ	(12)
Procedure	12,172	6,086–24,343	γ	(12)
Diagnosis	16,249	8,125–32,499	γ	(12)
Medicine	5,018	2,509–10,037	γ	(12)
Complications	15,070	7,535–30,140	γ	(12)
Ward	683	341–1,365	γ	(12)
Nursing	659	330–1,319	γ	(12)
Others	23,808	11,904–47,616	γ	(12)
Monthly cost of OMT (CNY)^a^	428	214–856	γ	(13)
Cost of HF hospitalization (CNY)^b^	10,926	5,463–21,852	γ	(13)
**Cost in scenario analysis**				
German MitraClip device cost	247,478	/	/	(10)
US MitraClip device cost	197,597	/	/	(14)
Japanese MitraClip device cost	179,504	/	/	(15)
UK MitraClip device cost	143,951	/	/	(16)
**Utility (Monthly)**				
NYHA I^c^	0.065	0.062–0.068	β	(17)
NYHA II	0.065	0.062–0.068	β	(17)
NYHA III	0.060	0.057–0.063	β	(17)
NYHA IV	0.055	0.052–0.058	β	(17)
Average disutility of complications	0.005	0.003–0.007	β	(10, 18)
Disutility of MitraClip procedure	0.043	0.034–0.051	β	(10, 19)
Disutility of HF hospitalization	0.10	0.08–0.13	β	(20, 21)
Discount rate	0.05	0–0.08	/	(22)

a Monthly cost of OMT is 428 = (679^*^29/2 + 711.1^*^19.2)/(29/2 + 19.2)^*^6.75^*^1.043 ^*^1.024^*^1.018^*^ 1.004/12.

b Cost of HF hospitalization is 10926 = (1218.4^*^36.7/2 + 1646.8^*^29.6) /(29.6 + 36.7/2)^*^6.75^*^1.043 ^*^1.024 ^*^1.018^*^1.004.

c Monthly utility of NYHA I is 0.065 = 0.780/12.

#### Utility

The utility of MitraClip-related cost was derived from a study of cost-effectiveness analysis, which reported that the one-month disutility for the MitraClip procedure was −0.043 (10, 19). The utility of different NYHA classifications was obtained from a study of the Chinese population (17). The utilities of NYHA I, II, III, and IV were 0.78, 0.78, 0.715, and 0.66, respectively. Regarding the utility of HF hospitalization, the common disutility of −0.1 was employed in the model (20, 21). Similar to the input of costs, the input of NYHA utilities was also converted to monthly utility, but other one-time utilities were not converted (Table 2).

### Analysis

The primary outcome of the study was the incremental cost-effectiveness ratio (ICER), which represented incremental costs per quality-adjusted life-year (QALY). The willingness-to-pay (WTP) threshold was set three times of per capita gross domestic product (GDP) in China in 2021, according to the China Guidelines for Pharmacoeconomic Evaluations (22), which was 242, 928 *CNY* = 80, 976 *CNY x* 3. MitraClip would be considered cost-effective if the ICER obtained was lower than the WTP threshold. Otherwise, it would be considered not cost-effective. Moreover, if MitraClip was not cost-effective, the cost-effective cost would be calculated, mainly including the overall cost and the cost of the MitraClip device. Scenario analysis based on the cost of the MitraClip device in other regions was also performed.

Sensitivity analysis included one-way sensitivity analysis and probabilistic sensitivity analysis (PSA). In the one-way sensitivity analysis, input parameters varied between their 95% confidence interval (CI), and the results of one-way sensitivity were shown with a Tornado Diagram. In the PSA, 10,000 times of Monte Carlo simulation based on probabilistic sensitivity sampling was employed. Costs were assumed to follow the gamma distribution. Transition probability and utility were assumed to follow the beta distribution in the PSA. The results of PSA were illustrated using a scatter plot and cost-effectiveness acceptability curve.

## Results

Table 3 shows model input values for baseline patient characteristics of the COAPT population.

**Table 3:** Model input values for baseline patient characteristics of the COAPT population.

**Parameters**	**COAPT population**
Age, years (mean)	72.3
Male (%)	64.1
Diabetes (%)	37.3
Hypertension (%)	80.5
Previous myocardial infarction (%)	51.5
Chronic obstructive pulmonary disease (%)	23.3
History of atrial fibrillation or flutter (%)	55.3
Body-mass index (kg/m^2^)	27.1
Anemia (%)	61.3
STS risk score	
Mean (%)	8.2
≥8% (%)	42.7
Cause of cardiomyopathy (%)	
Ischemic	60.8
Nonischemic	39.2
NYHA class (%)	
I	0.2
II	39.0
III	52.5
IV	8.3
Hospitalization for heart failure within previous 1 year (%)	57.2
Previous cardiac resynchronization therapy (%)	36.5
Previous implantation of defibrillator (%)	31.3
B-type natriuretic peptide level (pg/ml)	1016.0
N-terminal pro–B-type natriuretic peptide level (pg/ml)	5559.1
Severity of mitral regurgitation (%)	
Moderate-to-severe, grade 3+	52.2
Severe, grade 4+	47.9
Effective regurgitant orifice area (cm^2^)	0.41
Left ventricular end-systolic dimension (cm)	5.3
Left ventricular end-diastolic dimension (cm)	6.2
Left ventricular end-systolic volume (ml)	134.9
Left ventricular end-diastolic volume (ml)	192.7
Left ventricular ejection fraction	
Mean (%)	31.3
≤40% (%)	82.1
Right ventricular systolic pressure (mm Hg)	44.3

### Base case analysis

In the base case analysis, the lifetime cost for a patient in the MitraClip cohort was 423,817 CNY, and the lifetime cost in the OMT cohort was 28,369 CNY. The corresponding effectiveness in both cohorts was 2.32 QALY and 1.80 QALY, respectively. The incremental cost and increment effectiveness were 395,448 CNY and 0.52 QALY, respectively; thus, an ICER of 754,410 CNY/QALY was obtained. The ICER was higher than the WTP threshold of 242,928 CNY/QALY (Table 4).

**Table 4:** Base case analysis and scenario analysis.

	**Arm**	**Cost of MitraClip device (CNY)**	**Cost of overall MitraClip (CNY)**	**Lifetime cost (CNY)**	**Lifetime eff (QALY)**	**Incre cost (CNY)**	**Incre eff (QALY)**	**ICER** **(CNY/QALY)**	**ICER/WTP**
Base case	OMT	/	/	28,369	1.80	/	/	/	
	MitraClip	322,000	395,659	423,817	2.32	395,448	0.52	754,410	3.1
Scenario 1: German MitraClip device cost	MitraClip	247,478	321,137	349,295	2.32	320,926	0.52	612,241	2.5
Scenario 2: US MitraClip device cost	MitraClip	197,597	271,256	299,414	2.32	271,045	0.52	517,082	2.1
Scenario 3: Japanese MitraClip device cost	MitraClip	179,504	253,163	281,321	2.32	252,952	0.52	482,566	2.0
Scenario 4: UK MitraClip device cost	MitraClip	143,951	217,610	245,768	2.32	217,399	0.52	414,740	1.7
Scenario 5: Cost-effective cost 1	MitraClip	54,319	/	155,707	2.32	127,339	0.52	242,928	1
Scenario 6: Cost-effective cost 2	MitraClip	/	127,978	155,707	2.32	127,339	0.52	242,928	1

OMT, optimal medical therapy; CNY, Chinese Yuan; QALY, quality-adjusted life-year; Incre, incremental; Eff, effectiveness; ICER, incremental cost-effectiveness ratio; WTP, willingness-to-pay. Cost of overall MitraClip included the cost of the MitraClip device, procedure cost, nursing cost, ward cost, diagnosis cost, medicine cost, complication cost, and others.

In the lifetime simulation, an HF patient with secondary MR who received MitraClip would suffer approximately 1.16 HF hospitalizations, and it was 1.51 if OMT alone was given. Additionally, an HF patient who received MitraClip had a life expectancy of 3.72 life years, and it was 2.90 life years for those who received OMT alone.

### Scenario analysis

As shown in Table 4, the cost of the MitraClip device ranged from 143,951 CNY to 247,478 CNY in different regions, and the ICER based on these costs and the Chinese setting was always higher than the WTP threshold. When the MitraClip device cost was lower than 54,319 CNY (about 16.9% of the current price), or the overall cost of MitraClip was lower than 127,978 CNY (about 32.3% of the current cost), the ICER would be lower than the WTP threshold.

### Sensitivity analysis

**Figure 2:**
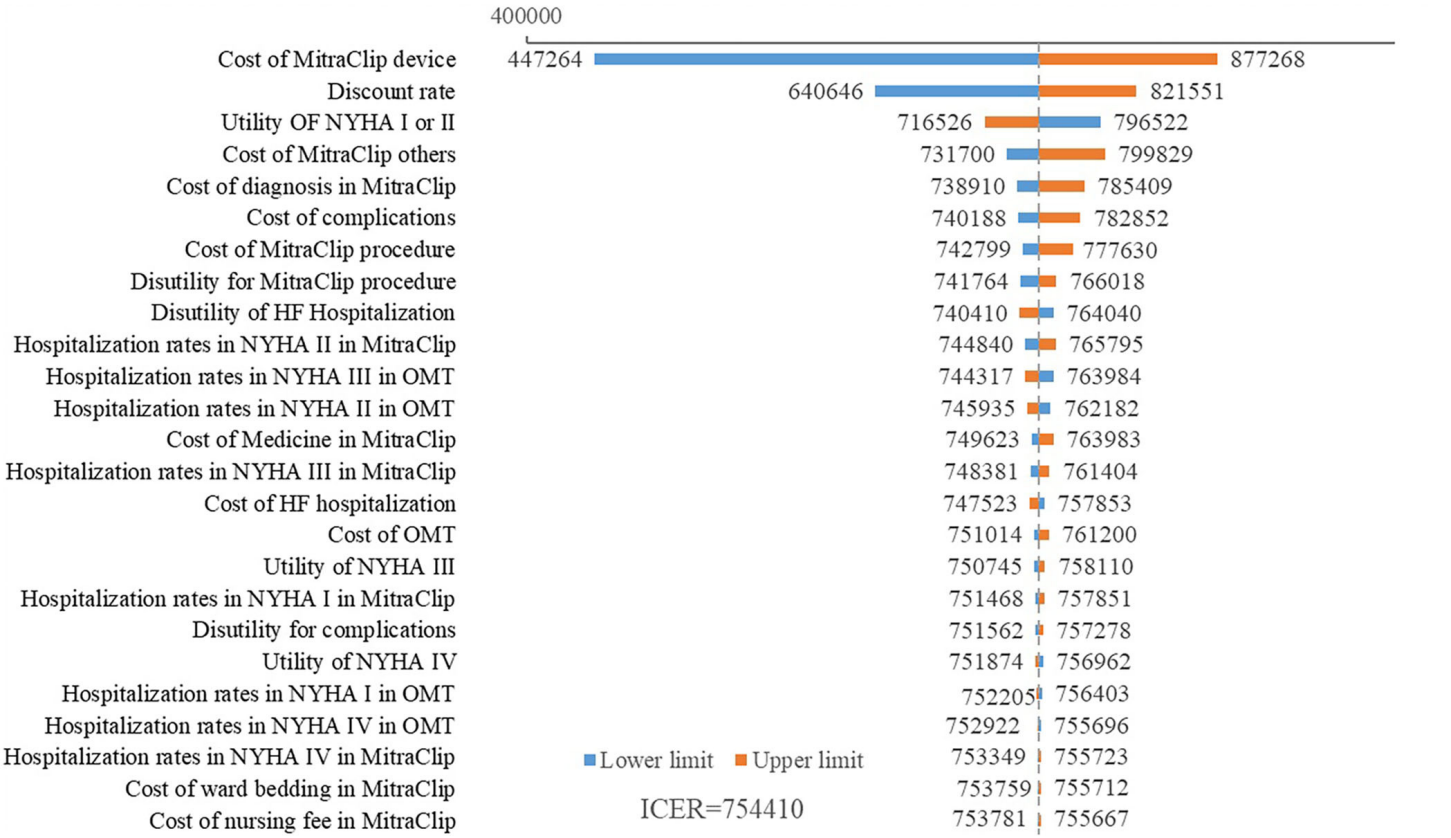
Tornado diagram of ICER based on different input parameters. The cost of the MitraClip device had the largest impact on the ICER fluctuation. The discount rate also impacted the ICER. Other parameters had little impact on the ICER.

One-way sensitivity analysis showed that the cost of the MitraClip device impacted most ICER fluctuations, and the discount rate impacted the ICER secondly. Whatever the cost of the MitraClip device or the discount rate ranged, the ICER was always higher than the WTP threshold (Figure 2).

**Figure 3:**
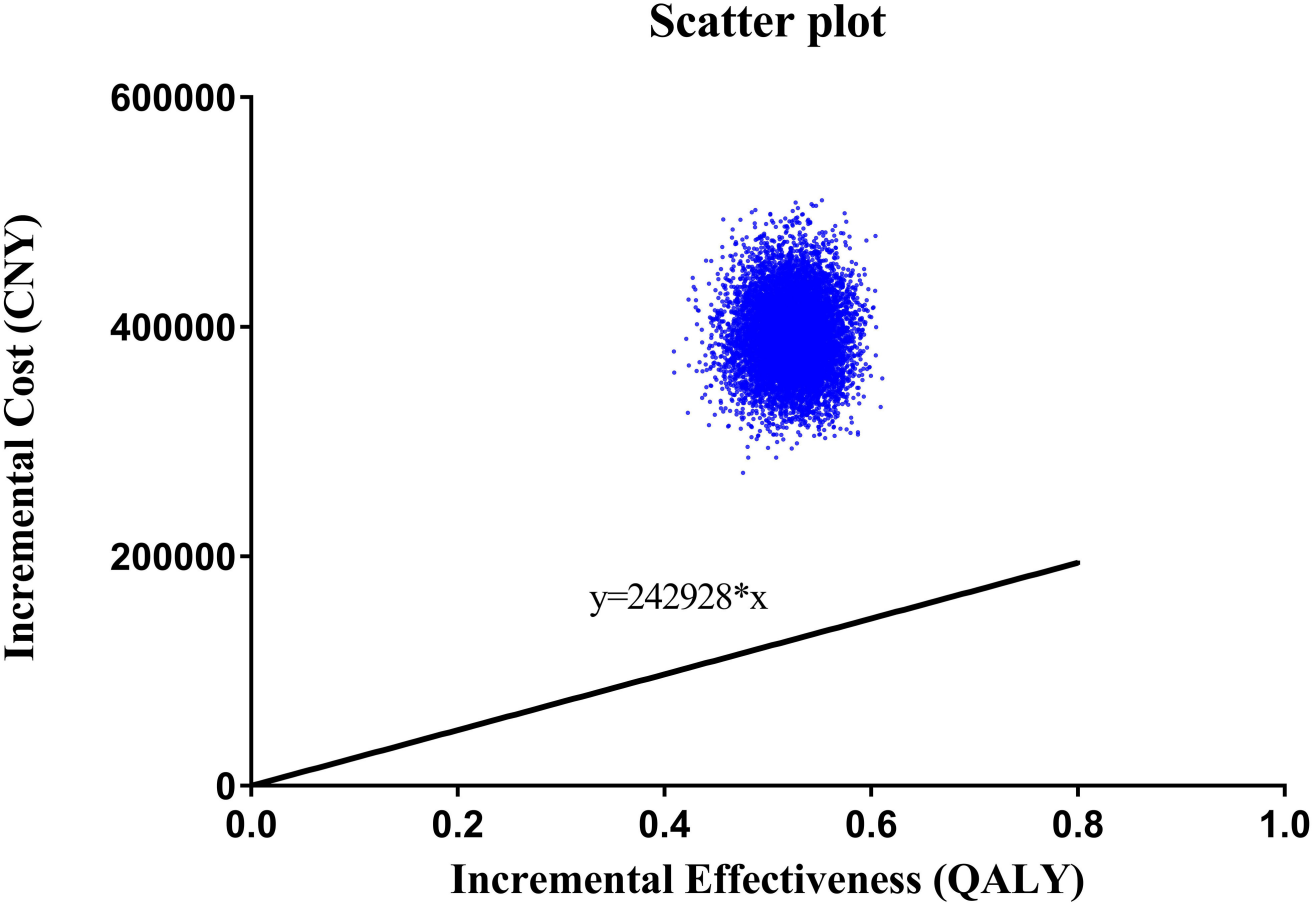
Scatter plot based on 10,000 times Monte Carlo simulation. The straight line indicated the WTP threshold. The dots were almost all above the line.

**Figure 4:**
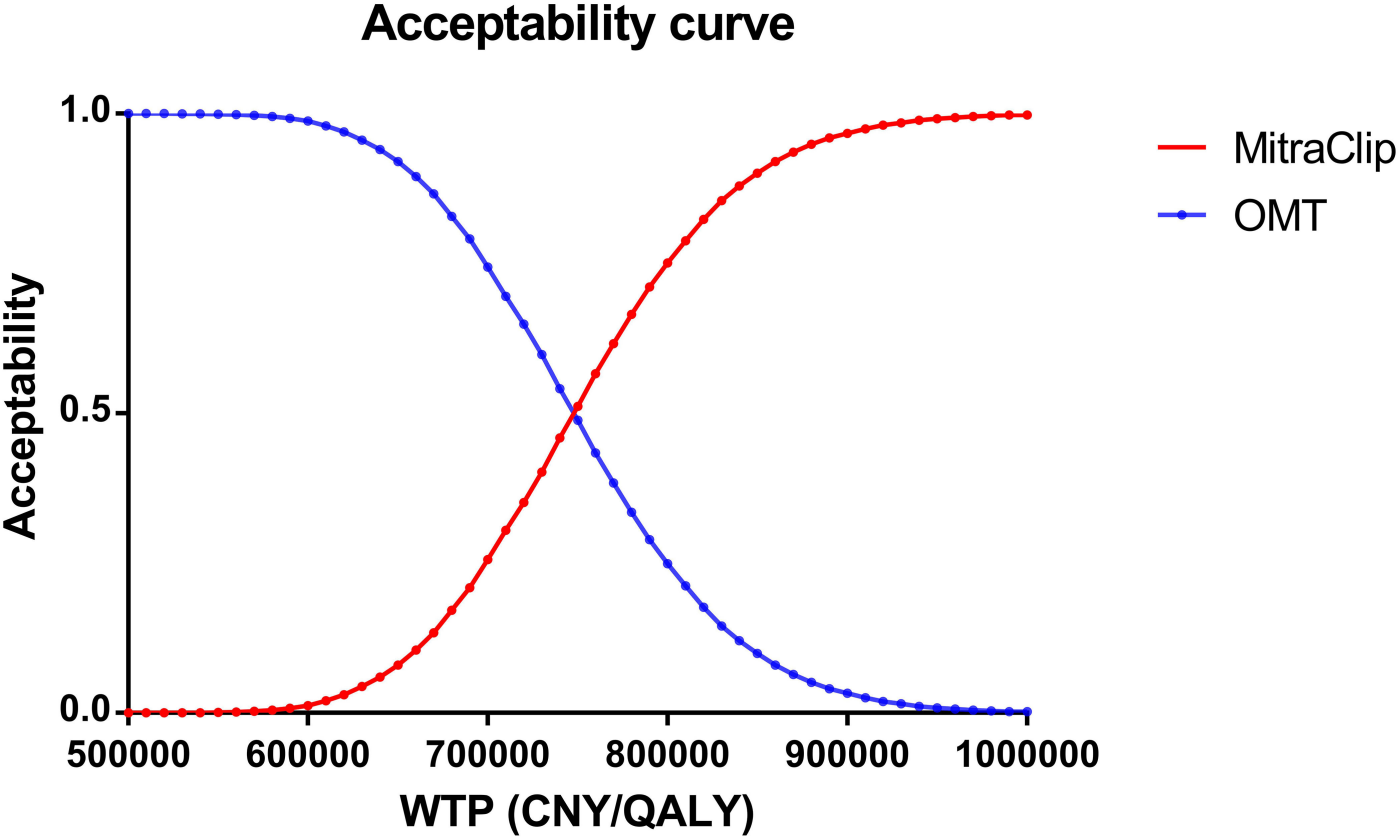
Cost-effectiveness acceptability curve based on probabilistic sensitivity analysis. The acceptability of MitraClip grew higher as the WTP threshold increased. When the WTP threshold was about 750,000 CNY/QALY, MitraClip shared similar acceptability with OMT.

A scatter plot based on PSA showed that under the WTP threshold of 242,928 CNY/QALY, there was a <1% probability that MitraClip was of cost-utility (Figure 3). Cost-utility acceptability curve showed that when the WTP threshold was about 750,000 CNY/QALY, MitraClip shared similar acceptability with OMT in Chinese patients (Figure 4).

## Discussion

The present study was the first one to investigate the cost-effectiveness of MitraClip in Chinese HF patients with secondary MR. In our analysis, we found that a patient treated with MitraClip could gain an additional 0.52 QALY than those treated with OMT, but the incremental cost was 395,448 CNY, causing an ICER of 754,410 CNY/QALY (equal to 116,963 USD/QALY), which is higher than the WTP threshold in China in 2021. MitraClip was considered not cost-effective in the current Chinese setting.

Three previous studies have tested the cost-effectiveness of MitraClip against OMT in the UK. One of the studies used data from the EVERSET II trial that included patients with primary and secondary MR and found that the ICER was £52,947 /QALY (equal to 469,956 CNY/QALY or 72,844 USD/QALY) (24). The second study based on the COAPT trial has reported an ICER of £30 057/QALY (equal to 266,785 CNY/QALY or 41,352 USD/QALY) (25). Another study also based on the COAPT trial has shown that the ICER of MitraClip was £23,270/QALY [equal to 206,544 CNY/QALY or 32,015 USD/QALY] (16). One study from Germany has shown that the MitraClip was cost-effective, with an ICER standing at €59,728 (equal to 455,736 CNY/QALY or 70,640 USD/QALY) (26). Additionally, MitraClip has been considered a cost-effective procedure in Italy (27). Almost all published papers have concluded that the obtained ICER ranged from 9,353 to 72,844 USD/QALY (24, 27). However, the ICER in our study was much higher than that in other studies. It might be attributed to the following aspects. First, the cost of overall MitraClip in China is higher than in other regions. According to our search of published articles, the cost of a MitraClip device ranged from 143,951 to 247,478 CNY in different countries (10, 16), but the price in China is 322,000 CNY, which is about twice the price abroad. Moreover, there is not so much difference in other MitraClip-related costs in China and other countries. Second, the cost of OMT in China is much lower than that in other regions (13), partly due to the collective purchasing policy launched by the Chinese government to provide better healthcare services. Third, the effectiveness of our study was lower than in other studies. The incremental effectiveness in Sakamaki's study was 1.44 QALY, but it was 0.52 QALY in our study mainly because their study was based on an observational study while our study was based on an RCT study (15). The incremental effectiveness in our study was almost consistent with Estler's one as we adopted the same model but was not completely consistent as the discount rate in China was higher than that in Germany (10).

As the largest developing country, China has 1.4 billion people, with 3.41% having MR (28), but the current cost of MitraClip is above the WTP threshold, which might partly account for the low proportion of Chinese HF patients with MR. Moreover, collective purchasing has decreased the cost of OMT in China, and novel agents, such as sodium-glucose cotransporter inhibitors and angiotensin receptor neprilysin inhibitors, have been widely used in Chinese HF patients and improved clinical outcomes (29). The ICER of MitraClip vs. OMT is 754,410 CNY/QALY, which is far higher than the WTP threshold of 242,928 CNY/QALY in China. Although the WTP threshold in some regions in China may be higher than that value due to the uneven economic development, the obtained ICER is still higher than the WTP threshold of the most developed regions in China. Additionally, we adopted the lowest cost abroad in our scenario analysis, and the ICER was still higher than the WTP threshold, suggesting the WTP threshold was lower in China than in other countries (10, 30). The deterministic analysis and uncertain analysis confirmed our findings. In our Tornado diagram, we found that the cost of MitraClip had the largest impact on the ICER fluctuation. However, although the 50% discount on the current price was adopted, the ICER was still higher than the WTP threshold. The cost-effectiveness acceptability curve indicated that the acceptability of MitraClip was < 1% under the current context.

Although MitraClip could benefit HF patients with MR, it is still not cost-effective in the current Chinese setting. One reason is that the MitraClip device was introduced to China in 2020, and the first MitraClip procedure was performed in 2021. Furthermore, the number of MitraClip procedures in China is not currently high. The Chinese government launched a collective purchasing policy in 2017 to lower the price of drugs, and medical services, drugs, or medical devices only with cost-effectiveness could be included in the purchasing lists and be purchased by Chinese public hospitals, which provided over 80% healthcare in China. MitraClip could be cost-effective only with a discount of 83% on the MitraClip device or a 68% discount on the overall cost.

Notably, our study was based on the COAPT study, demonstrating that MitraClip resulted in a lower HF hospitalization rate and lower all-cause mortality compared with OMT alone. However, the MITRA-FR proved that MitraClip did not improve the clinical outcomes compared with OMT (31). The main difference between the two studies lies in the population selection. In the COAPT study, enrolled patients had more severe MR, smaller LV end-diastolic volume, better guideline-directed medical therapy, and more experienced surgeons. Moreover, observational studies have also demonstrated that MitraClip entailed better survival outcomes compared with OMT (32, 33). These results suggested that the selection of proper patients is critical to clinical outcomes.

There were several limitations in our study. First, our study was performed based on validated mathematical models, and a real-world study might provide more powerful evidence, although one-way sensitivity analysis and PSA demonstrated the robustness of our results. Second, the cost of MitraClip was derived from an institution, which might not completely represent the real cost in China, and we resolved it by one-way sensitivity analysis using a 50% discount on the current price. Third, the transition probabilities were accessed from a published study and validated by authors but not from the raw data, which might have caused bias. Last, the study was performed from the perspective of a healthcare payer, and perhaps a perspective from society could offer more comprehensive information, but it was too difficult for us to finish it as we could not access the non-direct cost of MitraClip.

## Conclusion

In a lifetime simulation of MitraClip for HF treatment with secondary MR, MitraClip resulted in an additional 0.52 QALY in effectiveness and 395,448 CNY in cost compared with OMT. The ICER in the simulation was 754,410 CNY/QALY, which was higher than the WTP threshold in the current Chinese context. Thus, MitraClip was considered not cost-effective in Chinese HF patients with secondary MR.

## Data availability statement

The original contributions presented in the study are included in the article/Supplementary material, further inquiries can be directed to the corresponding authors.

## Author contributions

WX and KH collected data and drafted the manuscript, and YL came up with the idea and developed the model. All authors contributed to the article and approved the submitted version.

## Conflict of interest

The authors declare that the research was conducted in the absence of any commercial or financial relationships that could be construed as a potential conflict of interest.

## Publisher's note

All claims expressed in this article are solely those of the authors and do not necessarily represent those of their affiliated organizations, or those of the publisher, the editors and the reviewers. Any product that may be evaluated in this article, or claim that may be made by its manufacturer, is not guaranteed or endorsed by the publisher.
