# Supplementary material for: MitraClip for the treatment of heart failure with mitral regurgitation: A cost-effectiveness analysis in a Chinese setting
Source: Front Cardiovasc Med. 2022 Nov 11;9:970118. doi: 10.3389/fcvm.2022.970118 (PMC9691652; doi:10.3389/fcvm.2022.970118)
Supplement: Supplementary file 1 [file Table_1.DOC]

**Inclusion and exclusion criteria. (Same as those in the COAPT study)**

**Inclusion criteria (all must be present)**

1. Symptomatic secondary mitral regurgitation (3+ or 4+ by independent echocardiographic core laboratory assessment) due to cardiomyopathy of either ischemic or non-ischemic etiology

2. Subject has been adequately treated per applicable standards, including for coronary artery disease, LV dysfunction, mitral regurgitation and heart failure

3. NYHA functional class II, III or ambulatory IV

4. Subject has had at least one hospitalization for heart failure in the 12 months prior to enrollment and/or a corrected* BNP ≥300 pg/ml or a corrected NT-proBNP ≥1500 pg/ml 5. Local heart team has determined that MV surgery will not be offered as a treatment option, even if the subject is randomized to the Control group

6. Left ventricular ejection fraction ≥20% and ≤50%.

7. Left ventricular end-systolic dimension ≤70 mm

8. The primary regurgitant jet is non-commissural, and in the opinion of the MitraClip implanting investigator can be successfully be treated by the MitraClip (if a secondary jet exists, it must be considered clinically insignificant)

9. CK-MB obtained within prior 14 days is less than the local laboratory ULN

10. Transseptal catheterization and femoral vein access is feasible per the MitraClip implanting investigator

11. Age 18 years or older

12. Subject or guardian agrees to all provisions of the protocol, including the possibility of randomization to the Control group and returning for all required post-procedure follow-up visits, and has provided written informed consent

**Exclusion criteria (all must be absent)**

1. Untreated clinically significant coronary artery disease requiring revascularization

2. CABG, PCI or TAVR within the prior 30 days

3. Aortic or tricuspid valve disease requiring surgery or transcatheter intervention

4. COPD requiring continuous home oxygen therapy or chronic outpatient oral steroid use

5. Cerebrovascular accident within prior 30 days

6. Severe symptomatic carotid stenosis (>70% by ultrasound)

7. Carotid surgery or stenting within prior 30 days

8. ACC/AHA stage D heart failure

9. Presence of any of the following:

• Estimated PASP >70 mm Hg assessed by site based on echocardiography or right heart catheterization, unless active vasodilator therapy in the cath lab is able to reduce the 12 PVR to <3 Wood Units or between 3 and 4.5 Wood Units with v wave less than twice the mean of the PCWP

• Hypertrophic cardiomyopathy, restrictive cardiomyopathy, constrictive pericarditis, or any other structural heart disease causing heart failure other than dilated cardiomyopathy of either ischemic or non-ischemic etiology

• Infiltrative cardiomyopathies (e.g., amyloidosis, hemochromatosis, sarcoidosis)

10. Hemodynamic instability requiring inotropic support or mechanical heart assistance

11. Physical evidence of right-sided congestive heart failure with echocardiographic evidence of moderate or severe right ventricular dysfunction

12. Implant of CRT or CRT-D within the last 30 days

13. Mitral valve orifice area <4.0 cm2 by site-assessed TTE

14. Leaflet anatomy which may preclude MitraClip implantation, proper MitraClip positioning on the leaflets or sufficient reduction in mitral regurgitation by the MitraClip.

15. Hemodynamic instability defined as systolic pressure < 90 mmHg with or without afterload reduction, cardiogenic shock or the need for inotropic support or intra-aortic balloon pump or other hemodynamic support device.

16. Need for emergent or urgent surgery for any reason or any planned cardiac surgery within the next 12 months.

17. Life expectancy <12 months due to non-cardiac conditions

18. Modified Rankin Scale ≥4 disability.

19. Status 1 heart transplant or prior orthotopic heart transplantation

20. Prior mitral valve leaflet surgery or any currently implanted prosthetic mitral valve, or any prior transcatheter mitral valve procedure.

21. Echocardiographic evidence of intracardiac mass, thrombus or vegetation

22. Active endocarditis or active rheumatic heart disease or leaflets degenerated from rheumatic disease (i.e., noncompliant, perforated)

23. Active infections requiring current antibiotic therapy

24. Transesophageal echocardiography (TEE) is contraindicated or high risk

25. Known hypersensitivity or contraindication to procedural medications which cannot be adequately managed medically

26. Pregnant or planning pregnancy within next 12 months

27. Currently participating in an investigational drug or another device study that has not reached its primary endpoint.

28. Subject belongs to a vulnerable population or has any disorder that compromises his/her ability to give written informed consent and/or to comply with study procedures
